# Virtual surgical planning and 3D printing: Methodology and applications in veterinary oromaxillofacial surgery

**DOI:** 10.3389/fvets.2022.971318

**Published:** 2022-10-21

**Authors:** Jan R. S. Klasen, Graham P. Thatcher, Jason A. Bleedorn, Jason W. Soukup

**Affiliations:** ^1^Tierklinik Germersheim, Germersheim, Germany; ^2^Department of Surgical Sciences, School of Veterinary Medicine, University of Wisconsin-Madison, Madison, WI, United States

**Keywords:** oromaxillofacial surgery, computer-aided surgical planning, virtual surgical planning, veterinary, surgical guide, 3D printing

## Abstract

Virtual surgical planning is the process of planning and rehearsing a surgical procedure completely within the virtual environment on computer models. Virtual surgical planning and 3D printing is gaining popularity in veterinary oromaxillofacial surgery and are viable tools for the most basic to the most complex cases. These techniques can provide the surgeon with improved visualization and, thus, understanding of the patients' 3D anatomy. Virtual surgical planning is feasible in a clinical setting and may decrease surgical time and increase surgical accuracy. For example, pre-operative implant contouring on a 3D-printed model can save time during surgery; 3D-printed patient-specific implants and surgical guides help maintain normocclusion after mandibular reconstruction; and the presence of a haptic model in the operating room can improve surgical precision and safety. However, significant time and financial resources may need to be allocated for planning and production of surgical guides and implants. The objectives of this manuscript are to provide a description of the methods involved in virtual surgical planning and 3D printing as they apply to veterinary oromaxillofacial surgery and to highlight these concepts with the strategic use of examples. In addition, the advantages and disadvantages of the methods as well as the required software and equipment will be discussed.

## Introduction

Veterinary oromaxillofacial surgery (OMFS) is an ever-evolving field in which surgeons continue to advance novel approaches and improve patient outcomes. Coupled with this advancement is a client base that remains receptive to state-of-the-art interventions that may improve the lives of their pets. Such interventional efforts create significant challenges in surgical planning and execution. The OMF region is one with a high density of significant blood vessels, nerves, sensory organs (eyes, nasal cavity, inner and middle ear, and brain) and osseous structures critical to function. Lack of surgical precision can lead to complications with long term functional detriment to the patient (1). Minimal covering by soft tissues makes implant exposure and dehiscence more likely than in other regions of the body (2). Furthermore, OMF bone morphology is complex with many different planes and contours, as well as substantial variation in skull shape and size between and within species (3). Virtual surgical planning and 3D printing allows for surgical planning and rehearsal that has been shown to improve surgical precision and decrease intra-operative time (4). Three-dimensional printing has improved understanding of complex anatomy and pathology, improved surgical planning and allowed for improved pre-surgical preparation, particularly in the field of OMFS. The clinical application of 3D printing in veterinary OMFS has been well described (5, 6).

These challenges also provide opportunities for methodological and technological advancement. Custom, precise, and size-specific implants can help overcome this challenge by matching the unique contours in and around the OMF region. Surgeons also utilize technology to create individualized surgical plans, patient-specific guides, and implants (5, 7). The purpose of this manuscript is to provide a description of the principal elements of computer-aided surgical planning: imaging, segmentation and computer modeling, virtual surgical planning (VSP), design/manufacturing and 3D printing as it applies to companion OMFS.

## Imaging

All aspects of computer-aided surgical planning require use of high resolution images and the image modality selected will depend on considerations outline in Table 1 (8). Spatial resolution and contrast resolution are important considerations. Spatial resolution is the ability of an imaging modality to differentiate between two separate objects (9). For example, the distinction between the infraorbital canal and the surrounding maxilla. High spatial resolution image data is better suited for manual and automated image segmentation. Contrast resolution is the ability for an image modality to differentiate between adjacent image intensities. For example, the ability to differentiate areas of tumor contrast enhancement from surrounding tissues. Both conventional computed tomography (CT) and cone beam CT (CBCT) have high spatial resolution, but have varying and limited contrast resolution making these modalities the imaging of choice in hard tissue interventions that are typical of OMFS. CBCT has the advantages of ease of use, lower radiation exposure and affordability relative to conventional CT (10). However, its poor contrast resolution generally precludes its use for oncological and soft tissue surgical planning (11).

**Table 1:** Imaging modalities comparisons.

**Modality**	**Advantages**	**Disadvantages**	**Specifics**
CT	- Good soft tissue contrast - Good for contrast studies	- Anisotropic voxels - Limited resolution - Larger slice thickness	*Slice thickness*: 0.5–2.00 mm *Matrix:* Relatively high resolution resulting in small pixel and voxel sizes (0.3–0.625 mm) depending on FOV diameter *FOV:* 320–400 mm
CBCT	- High resolution - Small slice thickness	- Poor soft tissue contrast - Small FOV	*Slice thickness:* 0.1–0.4 mm *Matrix:* very high (pixel and voxel sizes 0.07–0.3 mm) *FOV:* 130–210 mm
MRI	- Ideal for soft tissue contrast - No radiation	- Challenging image segmentation - Not suited for bone - Long acquisition time	*Slice thickness:* ~ 2–5 mm *Matrix:* small matrix compared to CT *FOV:* up to 500 mm *SNR:* varies greatly depending on acquisition time, slice thickness and matrix 0.5–3 T magnetic field strength
3D Scanner	- Excellent resolution - Easy and fast - Color gradients obtained - No radiation	- Only surface scanning - Poor for wet and glossy surfaces - Limited anatomical access due to scanner size	*Resolution:* 10–30 μm *Acquisition time:* 30–120 sec

*CT*, computed tomography; *CBCT*, cone beam computed tomography; *MRI*, magnetic resonance imaging.

The desire for thin slices to maximize anatomical detail is often balanced with the demand placed on the imaging equipment and the desire to minimize radiation exposure. While a minimum resolution is not agreed upon, CT scans that maximize resolution and use bone algorithms and thin image slices are recommended for OMF cases. The quality limiting step of medical 3D printing is image resolution, which is often between 0.1 and 0.5 mm for most CT and CBCT scanners (12). Most commercial grade 3D printers can print with resolution as low as 50 μm (13). Therefore, every effort should be made to optimize data acquisition. Slice thickness should always be the thinnest possible and resolution should be the highest if possible. Authors found results less satisfactory when slice thickness exceeded 1.25 mm. Volumetric renderings lose fidelity with patient anatomy as slice thickness increases (14). A high matrix resolution is preferred, especially when a large field of view is being used. A high matrix size can be 2048 x 2,048 pixels in a 320 mm Field of View. This means that the area a CT Scanner can cover is 320 x 320 mm and the resolution within this Field of View is 2,048 x 2,048 pixels. The theoretical size of one pixel would be 0.156 mm in this example. A lower matrix size would be 512 x 512 pixels. Most CT Scanners cannot change their matrix size and many conventional CT Scanners have matrix sizes of 512 x 512 or 1,024 x 1,024. A CT Scanner with a high matrix size but a small Field of View can have a sufficient resolution as pixel size is small. As these settings are usually fixed it is nothing a clinician can easily change but one should know matrix size and Field of View can be quality limiting factors in data acquisition (9). Although there are disadvantages regarding the signal to noise ratio, a higher matrix resolution and, therefore, the maximum available spatial resolution of the CT scanner is desired. Additionally, in cases where delineation of pathology from normal tissues requires contrast studies (e.g., OMF tumors), conventional CT is currently preferable over CBCT as contrast resolution is still far superior (15).

Magnetic resonance imaging (MRI) data can also be used to generate 3D computer models and 3D prints. Typical clinical high-field scanners are 1.5–3 Tesla which offer improved soft tissue detail but often larger slice thickness (2–5 mm) (16). Model generation can be challenging as segmentation processes are less automated and require greater operator skill and time. Although resource intensive, combining, and overlying CT and MRI data can provide complementary information. Higher powered (7 Tesla) research MRI can produce the sub-millimeter slice interval detail required for OMFS (16). However, they are currently prohibitively expensive and not widely available in veterinary medicine.

Three-dimensional scanning, like intraoral laser scanners that use structured light to scan surfaces, provides another opportunity for imaging in cases that require meticulous detail, namely orthodontics and orthognathic surgery cases (17). This technique is very much like a digital version of silicone impressions. Patients may require sedation or general anesthesia for scanning; however, the technique is non-invasive, is free from radiation exposure and provides superior resolution to CT/CBCT/MRI (17). Only surfaces can be scanned, but current scanners have excellent resolution which can produce high fidelity models with little post-processing. Although not widely used yet in veterinary patients, they may play a future role for taking impressions and fabrication of dental prostheses and orthodontic appliances. In human dentistry, 3D scanners have an established role in acquiring anatomic data of the patient's teeth and oral tissue (17). Impressions that were made in a conventional way are already being scanned at dental labs for integration into their digital workflow (18, 19).

## Data acquisition

**Figure 1:**
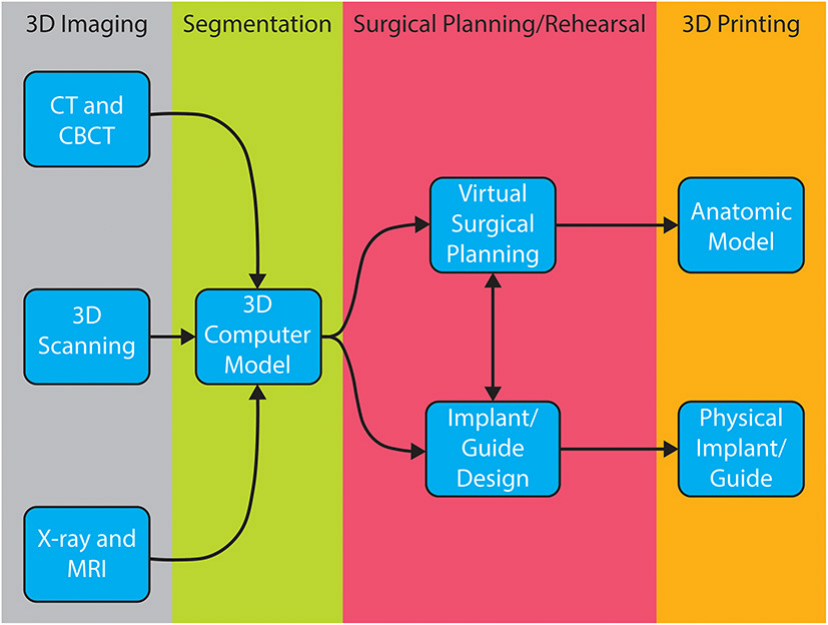
Overview of workflow from imaging to 3D printed model/implant.

Successful surgical planning is highly dependent on the ability of the dataset to accurately duplicate anatomic detail, the dataset to be translated into a computer model, and the software to facilitate surgical planning. Datasets are stored in digital imaging and communications in medicine (DICOM) format, which allows for universal sharing between hardware and software systems. Processing of DICOM data into 3D objects allows the operator to manipulate patient anatomy in a variety of software programs (20). After creation of the computer model, further manipulation allows for surgical planning and exportation of the file, typically as a Standard Tessellation Language (STL) file. A wide variety of software is available to the veterinary surgeon who wishes to pursue in-house VSP and/or 3D printing (Table 2). The overview of steps required to process image files to generate 3D models and implants is shown in Figure 1. Surgeons wishing to pursue implant 3D printing in-house should be prepared for the substantial time, effort, and financial investment. There are increasing numbers of commercial vendors to outsource all steps of this process. Several companies now offer 3D printing of bones and other anatomic structures. Careful consideration of the cost: benefit analysis for this technology is important when considering in-house or outsourcing 3D printing.

**Table 2:** Imaging processing and design software comparisons.

	**Best for**	**Advantages**	**Disadvantages**	**Learning curve**
Materialise Mimics	Advanced segmentation; VSP	Segmentation; VSP and simulation in one	High expense	++
InVesalius	Basic segmentation	Ease of use; open source	Thresholding only	+
Proplan CMF	Advanced segmentation; VSP	CMF/OMFS focus	Currently not sold to veterinarians	
Itk Snap	Intermediate segmentation	Open source	Underdeveloped user interface	+
3D Slicer	Advanced segmentation	Open source	Steep learning curve	+++
Materialise 3-Matic	Advanced modeling; VSP	Seamless integration with Mimics	Steep learning curve; high expense	+++
Geomagic FreeForm	Advanced modeling	Ideal for organic shapes	Steep learning curve; not ideal for non-organic shapes; high expense	++++
Solidworks	Parametric shapes; Advanced modeling	Very good for plate designs	Not ideal for non-organic shapes; high expense	+
Fusion 360	Intermediate modeling	Low expense	Not ideal for organic shapes	+
Meshmixer	Basic modeling	Open source	Very basic software; no precise modeling	++
Strata Sculpt	Advanced modeling with virtual clay	Affordable, good for organic shapes	No precise modeling; not ideal for geometric shapes	++

## Segmentation and computer modeling

The first image processing step is importation of DICOM data to a 3D modeling software. The region of interest (ROI) must be digitally partitioned from the surrounding tissue *via* a process known as segmentation. The goal of segmentation is to identify and separate the ROIs into individual components that are easier to identify and manipulate digitally.

**Figure 2:**
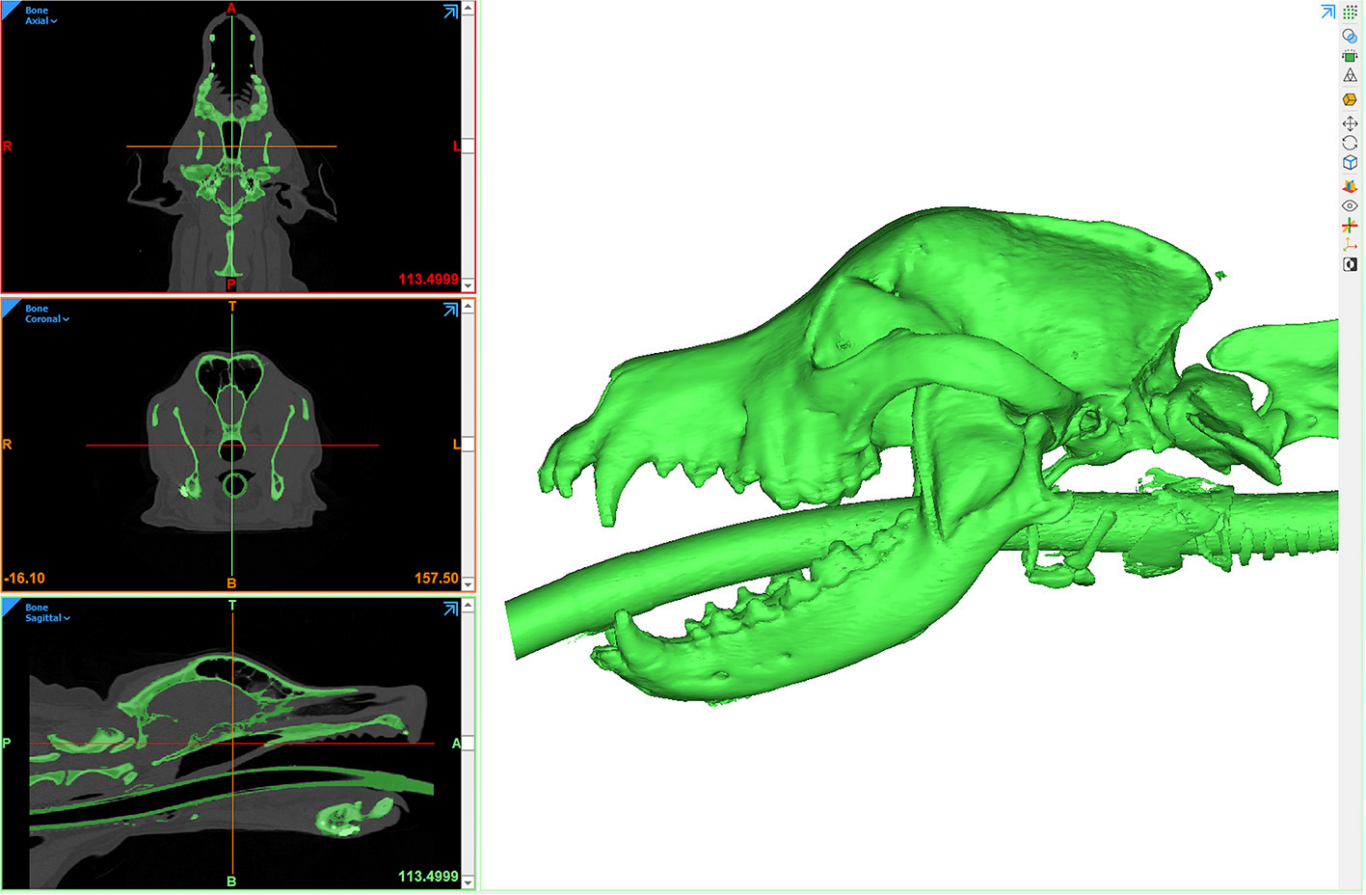
Computer screenshot depicting an automated thresholding process for segmentation and 3D computer model generation.

**Figure 3:**
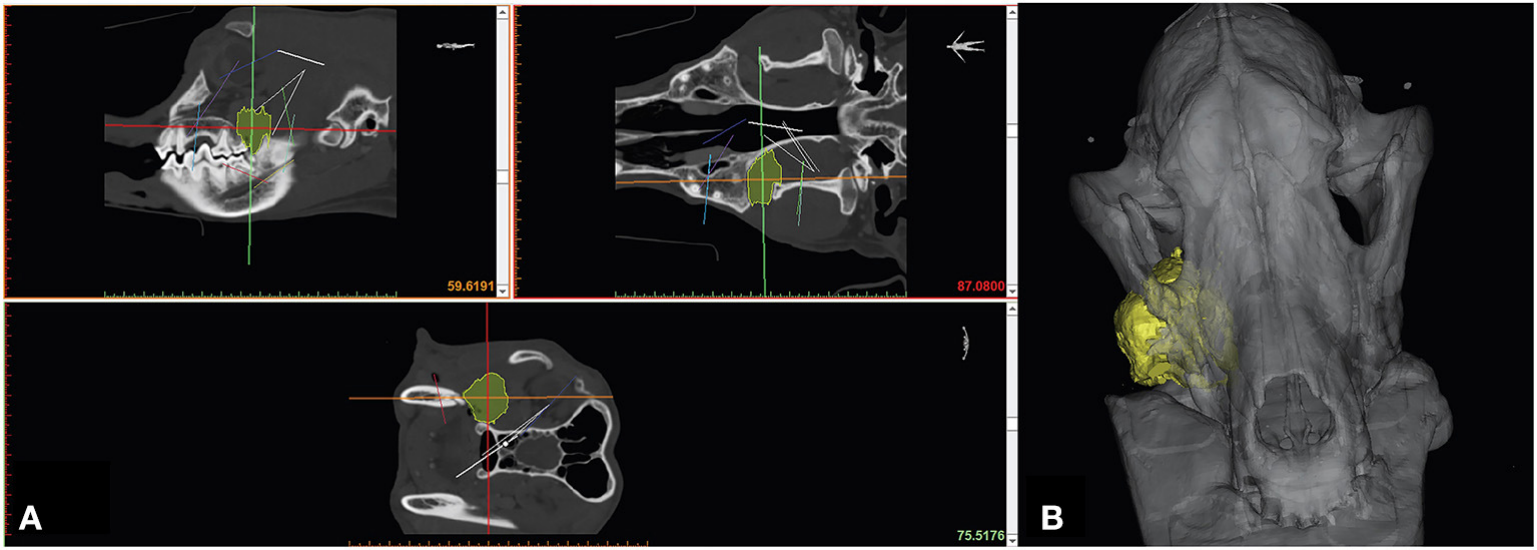
Manual segmentation of soft tissue neoplasm **(A)** with resultant 3D computer model **(B)**.

Segmentation can be performed in a variety of ways but typically involves some form of automated algorithm to reduce manual workload and time. Generally, segmentation begins with a semi-automated process of identifying and assigning pixels to a group based on a range of Hounsfield units (HU). A popular semi-automated algorithm is *volume growing* which uses thresholding to detect an anatomic structure within a certain range of HU and then saves it as one structure separated from the rest of the DICOM dataset. During the thresholding process the user defines a range of HU of the anatomic structure that is to be separated from the surrounding tissues. The software then automatically detects areas in every image where the HU of the tissue is within the specified HU range and connects them to one object (Figure 2). This information is generated for every slice of a CT/MRI scan and is then combined to create a 3D volume of the anatomic structure. Automation of mineralized tissue is well suited due to the extremely high HU in relation to surrounding soft tissues. Additionally, contrast-enhancing tumors can be separated but may require additional manual segmentation efforts and time (Figure 3). The selection of the adequate HU range is essential as improper assignment can result in anatomical components being artificially larger or smaller, which can result in improper surgical margins and poorly fitting osteotomy guides/implants. Due to the wide variance in pixel density distribution in a conventional CT dataset, the product of this segmentation step typically requires at least some manual refinement. The number of manual manipulation tools available is exhaustive and differs between various software programs. Artifacts that result from the segmentation or the 3D imaging (metal implants, etc.), referred to as beam hardening, itself must be digitally removed or smoothed out (21). The challenge lies in only removing the artifacts while maintaining fidelity to the patient's anatomy as mistakes at this stage may also lead to poorly fitting osteotomy guides/implants.

In many cases, particularly when planning osseous reconstruction after tumor resection, it is useful to mirror the healthy contralateral side of the patient onto the pathologic side. This allows the operator to print an anatomically correct “healthy” avatar that can be used to design, size, or pre-contour an appropriate implant. At this stage 3D mesh (STL) files of target anatomy can be exported for 3D printing if no other design work or data manipulation is needed. The STL file is then imported into a software program to set up the printing process. Print resolution and layer height [for fused deposition modeling (FDM) printers] are important factors that impact the surface quality. Density or infill will change the weight and structural integrity of the part. For many printing technologies support structures are required to keep the part in position during the printing process. At the same time, it is important to design the support structures for ease of removal without compromising the desired part. All these factors have an impact on printing time. Post-processing steps (and time) vary by printer type, but may include removal of support structures, rinsing off uncured surface resin, curing and/or drying resin, applying surface finishes and sterilizing.

## Surgical planning

The simplest approach to surgical planning is the use of outsourced 3D prints for surgical rehearsal. An intermediate approach is converting imaging data to models using 3D software and printing models in-house. Finally, the most complex endeavor is planning and performing virtual surgical correction on the computer (VSP) and subsequently printing the resultant anatomic models and designed components.

### Surgical rehearsal using 3D-printed anatomic models

**Figure 4:**
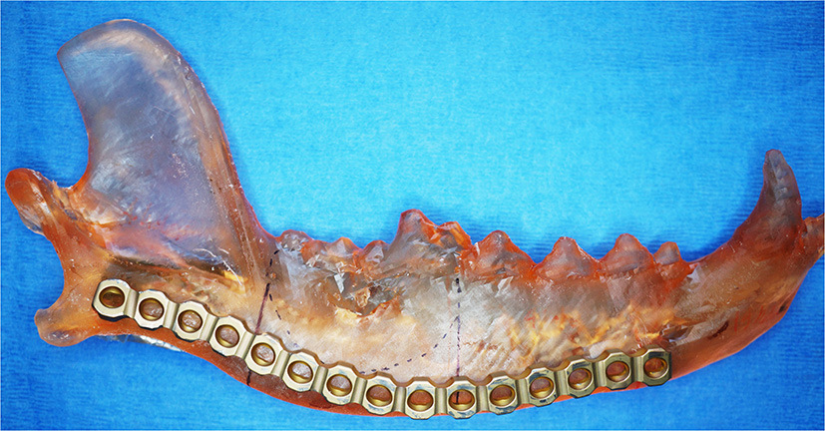
3D printed mandible used to pre-contour a mandibular titanium reconstruction plate. Reprinted from Thatcher GP, Soukup JW. Virtual surgical planning and 3D printing in veterinary dentistry and oromaxillofacial surgery. *Vet Clin North Am Small Anim Pract*. 2022; 52(1): 221–234 with permission from Elsevier.

A quick and accessible approach to using 3D printing in surgery is to print patient-specific models of the anatomic ROI. For this method, imaging data must be processed as previously described to generate a 3D volume mesh (STL file). Models can be printed in heat-stable materials on relatively low cost Fused Deposition Modeling (FDM) or Stereolithography (SLA) printers and sterilized for use in the operating room. This entire process can be performed in-house with relevant software and printers, or it may be completely outsourced. Surgical planning can be improved with an increased 3D understanding of the anatomic region and any pathology present (22). Appropriately sized implants can be selected and pre-contoured on full scale printed models (Figure 4). Anatomic models of most materials can be cut or drilled to simulate the surgical execution and provide haptic feedback for the surgeon (5). This relatively simple process can allow the surgeon to critically evaluate a proposed surgical plan, anticipate complications, reduce intraoperative decision-making, improve fit precision of commercial implants, and build confidence in the execution (22).

### Virtual surgical planning

Virtual surgical planning is a mechanism by which a surgery is planned and rehearsed completely within a virtual environment on the computer. Due to the complexity of the OMF region, it has become a rapidly utilized process in OMFS (23). Surgical resection of large tumors of the midface, periorbital and zygomatic regions are challenging and may require osteotomies often performed without direct visualization of the tumor and/or critical anatomical regions (e.g., maxillary artery, bulla, sinuses, and cranial vault). Rehearsing surgeries, where tolerances for mistakes may be in the submillimeter range, in a virtual environment may allow for resections to be performed with higher precision, accuracy and confidence. Precision and accuracy may be further increased with implementation of patient-specific surgical guides where vital structures can be accounted for in the design (24). Importantly, VSP requires substantial training and has a steep learning curve compared to benchtop rehearsal on 3D-printed models. It is important to consider building in workflows for software planning and design in a time effective manner. Collaborating with engineers proficient in CAD and medical imaging can improve tool workflow and balancing the clinical constraints for an individual case.

**Figure 5:**
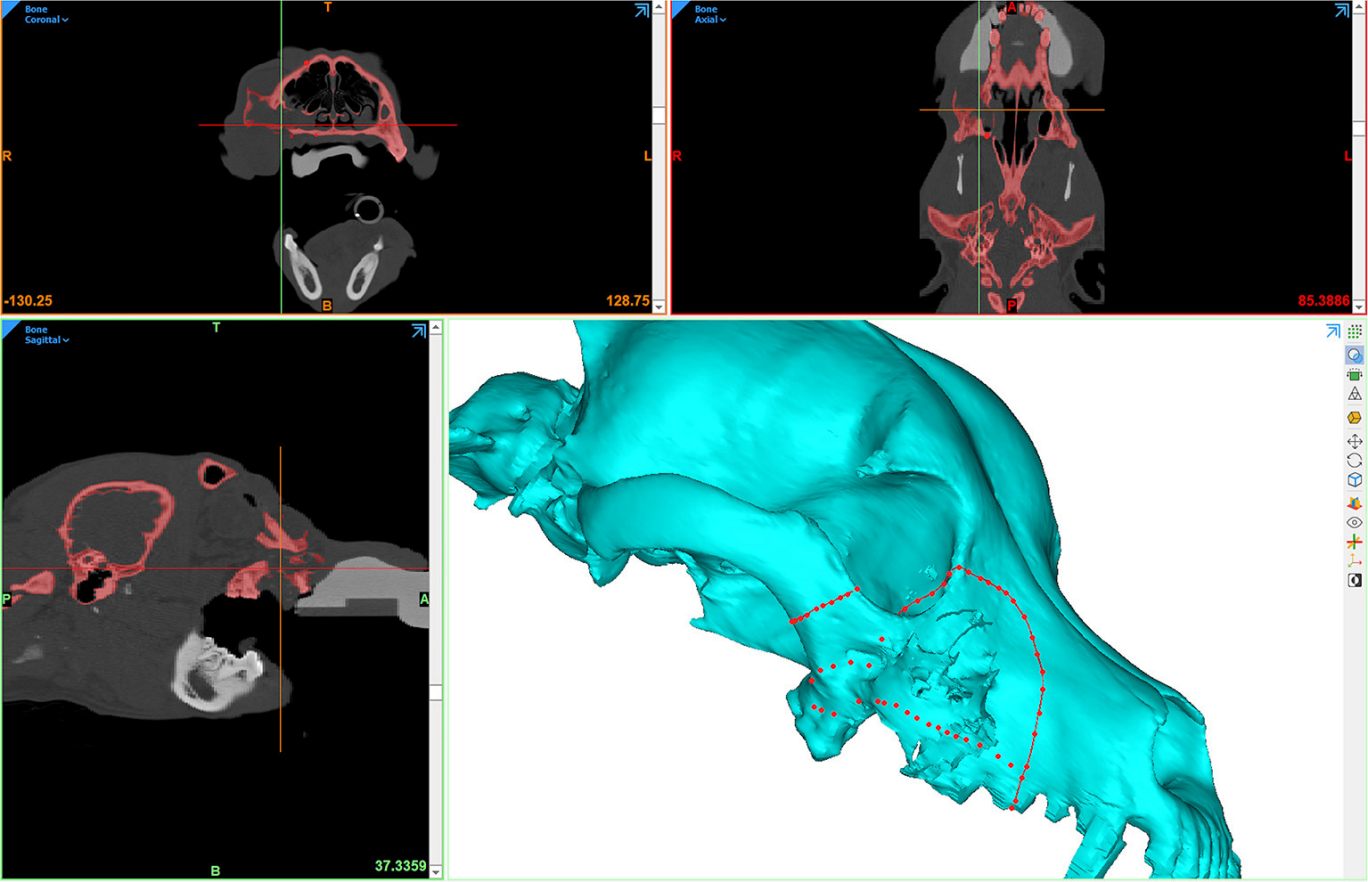
Computer screenshot depicting VSP in a patient with a caudal maxillary neoplasm. The red lines depict intended surgical margins, which are used to create virtual osteotomies.

Recent studies in humans demonstrated an improvement in obtaining tumor free surgical margins in the maxilla when VSP was compared to an optical guidance system based on anatomical landmarks in the CT scan or conventional planning by discussing the CT scan before surgery (25, 26). Human mandibular osteotomies and reconstructions based on 3D models were shown to be more accurate compared to using traditional x-ray-based planning (27–29). The use of 3D models and guides in humans have demonstrated the ability to decrease surgical time and minimize complications in mandibular reconstruction after tumor resection or trauma (29–31). These authors have successfully used VSP to plan and rehearse complex tumor resections of the orbitozygomaticomaxillary complex and for midface reconstructions (Figure 5). Simulating the entire surgery virtually allows for exploring endless variations, comparing various techniques, without the time and resources required to create a 3D print for each novel simulation. Combining VSP with surgical rehearsal on 3D printed models offers the benefits of both approaches for the most complex cases and is generally the preferred approach used by these authors (31, 32).

## Design and manufacturing

An important component of surgical planning is the design of surgical aids such as drill or osteotomy guides and the design of custom implants. One of the most crucial aspects of this design phase is the communication between designer and surgeon. It should be clear to both parties that it takes time to effectively work together, and a tight schedule usually cannot be met when working together for the first time. Inefficiencies lead to undesired results and a substantial increase in the time required to complete the design and manufacturing but may be minimized when the designer and the surgeon are the same person (33).

**Figure 6:**
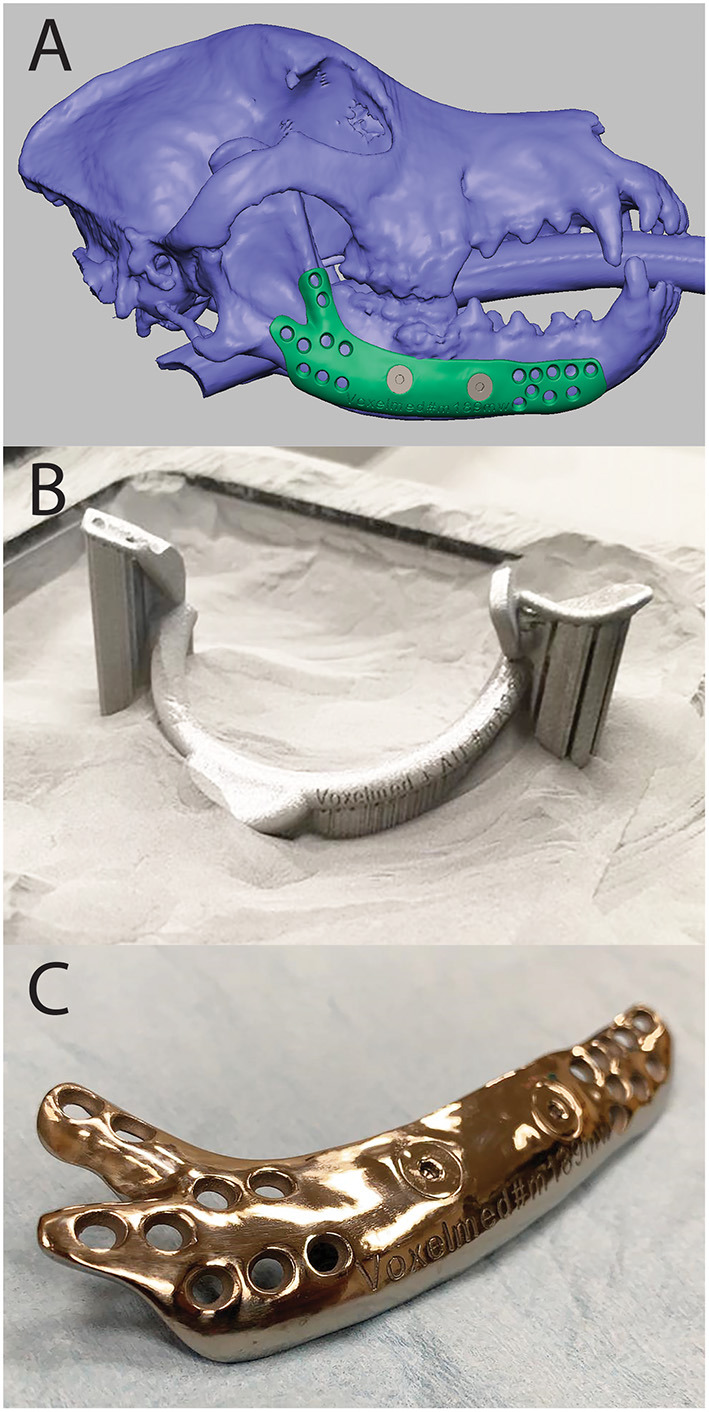
Computer Aided Design (CAD) of a mandibular implant shown in FreeForm Plus (3D-Systems, Rock-Hill, USA) **(A)**, 3D printed surgical guide for mandibulectomy and reconstruction in the print bed **(B)**, and 3D printed, patient-specific titanium mandibular reconstruction implant after post-processing **(C)**.

The first design is usually a rough sketch based on the specifications from the surgeon. The design evolves in an iterative process until the requirements for the patient and type of surgery are met. This usually involves sharing images of the design sketches and collaboration between designer and surgeon. 3D models can help to speed up this process but requires the surgeon to at least have the software and knowledge to view STL files (Figure 6). Material choices should be made early on as it will influence several decisions throughout the design phase. The timeline for an individual case may dictate the number of design iterations and/or selection of print materials (dry benchtop rehearsal vs. sterile surgery). If there is enough time before the scheduled procedure, performing simulated osteotomies on 3D-printed models and providing feedback to the engineer prior to finalizing the design, if necessary, may be beneficial. Models and guides for surgical rehearsals can be printed in draft or prototyping materials which is more cost effective and efficient.

## 3D printing

The process of 3D printing allows for the possibility of bringing objects from the virtual to the physical world in a cost and time effective manner. 3D printing usually is the manufacturing method most suited for personalized, low quantity, and/or complex objects. Although there are many types of 3D printing technologies, the most important and common polymer 3D printing processes used in veterinary medicine are FDM, SLA, and Selective Laser Melting (SLM). Each technique creates objects by subsequently creating layer after layer of the desired object until the final object has been created. Details and comparisons of printing technologies are provided in Table 3.

**Table 3:** 3D-printing methods comparisons.

	**Process**	**Advantages**	**Disadvantages**	**Brands**	**Expense**
Fused deposition modeling (FDM)	Extrusion of hot plastic	Low expense	Support structures needed unless multi extrusion; soluble supports	Prusa; Ultimaker; MakerBot	+
Stereolithography (SLA)	Selective curing of resin	High precision	Involved cleaning process; support structures needed	Formlabs; Anycubic	++
Selective LASER SINTERINg (SLS)	Selective melting of plastic powder	Design freedom due to lack of support structures	No support structures	Sinterit; 3DSystems	+++
Multi Jet Fusion (MJF)	Multi jet fusion	Full color; fast; no support structures	High expense	HP	++++
Selective laser melting (SLM)	Selective laser melting of metal powder	Many biocompatible metals can be printed; better surface quality compared to EBM	High expense; specially trained personnel needed	SLM-Solutions; EOS	+++++
Electron beam melting (EBM)	Selective electron beam melting of metal powder	Many biocompatible metals can be printed; better suited for high volume prints	High expense; specially trained personnel needed	Arcam (GE)	++++++

## Surgical guides

**Figure 7:**
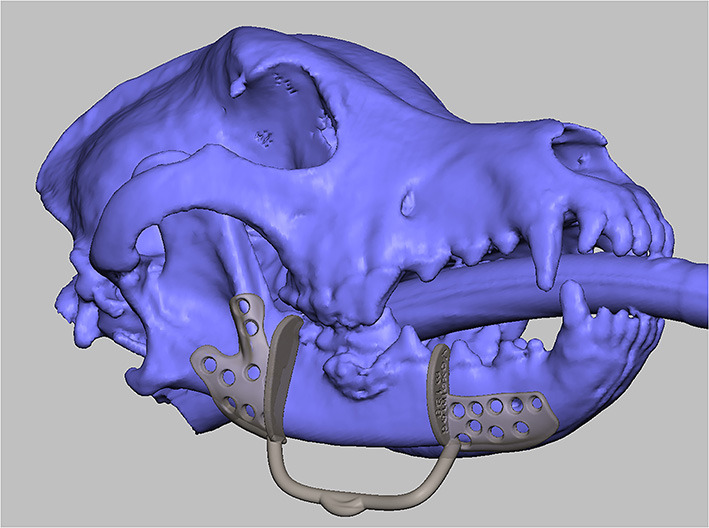
3D model of a surgical guide for mandibulectomy and reconstruction.

Patient-specific surgical guides allow for direct translation of a complex surgical plan into a precise operating room execution (Figure 7). Guides are typically designed to fit on a precise contour of the patient anatomy, and it is critical this location matches the location of the plan to achieve the desired trajectory. Guide design greatly depends on the type and size of the instrument a surgeon will use to make an osteotomy or drill path. Design should accommodate a tolerance for the instrument to avoid abrasion of the guide and formation of wear debris. Surgical guides are usually made from biocompatible polymers or sometimes metals (Figure 6). Osteotomies may be made using oscillating saws, dental drills or piezotomes which may require different guide design. As there are numerous different saw blades, burs, and piezo attachments the surgeon should let the designer know the exact model of the instrument in case it can be found in a design library (34). If it cannot be found it is crucial to take precise measurements of the instrument with a caliper or similar tool. Lack of this information will result in a less precise guide and, consequently, poorly placed pin or osteotomy. The highest precision osteotomies can be achieved using a Piezotome (34). Together with a surgical guide, the technique can be further improved (35).

Surgical guides should be printed using biocompatible materials and be able to withstand the forces of the instruments. If the anatomic location allows cooling channels for irrigation can be incorporated into the design of the guide. In addition to osteotomy guides, drill guides can be used to make sure important anatomical structures will be preserved (36). Alternatively, the final implant can be used as the drill guide. This is especially suited when using metal implants as other materials are more prone to damage by the drill. In addition, manufacturer provided drill sleeves may be connected to custom implants to act as a drill guide.

## Metal implants

Internal fixation plates come with the challenge that they need to be strong enough to withstand the forces of normal function, while minimizing weight and foreign material in the body. Importantly, preservation of local vital structures and minimization of soft tissue irritation must be considered. Patient-specific implants are typically designed to reconstruct the patient's defect to re-establish the normal anatomy. Implants are most often made from medical grade metals such as titanium, stainless steel, or cobalt chromium and, occasionally, ceramics (Figure 6). Custom implants are often used with commercially available bone screws; therefore, the type and exact dimensions of the screws need to be well understood by surgeon and designer. A current challenge is 3D printing bone plates for threaded locking screws to engage with. High precision 3D printing or special alloy combinations can be utilized to use locking screws with 3D printed plates. Conventional metal 3D printing of fine threads often come with less locking strength compared to machined locking mechanisms. The threads in current commercially available locking plate systems are typically machined after plate production. Additional machining after 3D printing prolongs the overall production process and adds cost. Ultimately, clear communication of all aspects of the final design should be discussed prior to production to avoid repeat printing and ensure a successful final product (37, 38).

## Limitations

While computer-aided surgical planning and 3D printing offers many substantial advantages, there are some limitations worth considering. Because the process of moving from CT data to a surgical outcome requires numerous steps, a mistake made early on can cause amplified problems much later in the process. The effort needed to correct for a mistake that was noticed downstream may be costly and time consuming. Acquisition of high-resolution CT data that accurately simulates the anatomy of the patient is critical. Therefore, poor choices made in imaging modality and imaging technique can lead to suboptimal outcomes. Mistakes in early processing steps (e.g., segmentation) can magnify the degree of anatomical infidelity of the 3D models and/or implants leading to poor model or guide fit. Care and time must be taken to get an accurate segmentation outcome. Manual refinement of the segmentation process can take substantial amounts of time in complex cases. The amount of time needed is proportional to the operator's familiarity with the software environments, which often have significant learning curves. However, time invested into pre-surgical planning will lead to decreased intra-operative time and likely better surgical outcomes.

Additional drawbacks to VSP and 3D printing include increased time (planning, communication, manufacturing) and costs (software, production, salary) (30). The time from imaging data acquisition to the surgical table will depend not only on the ability to efficiently navigate the modeling and design software but will also depend on details surrounding the 3D printing process. In-house 3D printing is labor and cost intensive but may offer more rapid turnaround (~24–48 hours) compared to outsourcing (days–weeks). The costs of desktop quality SLA resin printers have come down significantly in the last decade and these are becoming common place in the armament of the veterinary OMF surgeon. Patient specific implants are typically outsourced to companies with industrial metal printers as they require a substantially higher investment.

## Case examples

### Case 1—VSP and 3D printed patient model for implant contour

**Figure 8:**
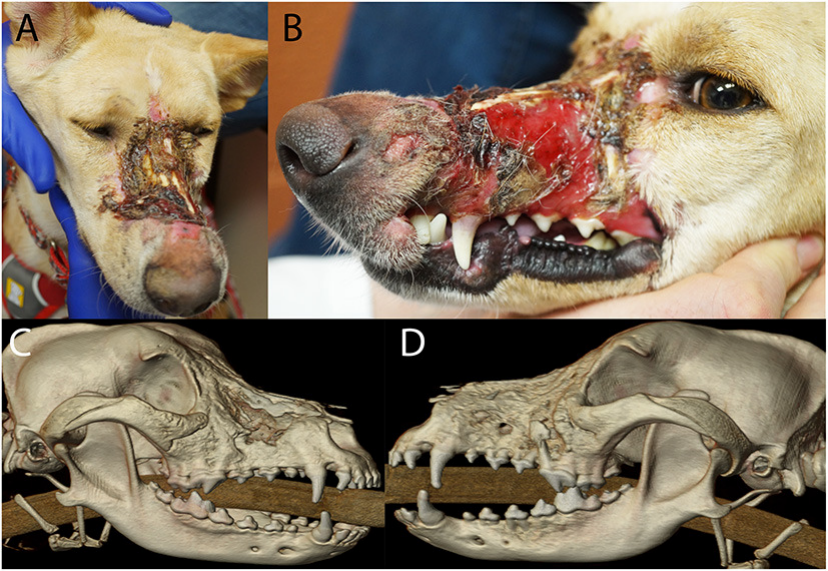
Photographs **(A,B)** of the soft tissue damage and volumetric renderings of the right **(C)** and left **(D)** sides depicting the hard tissue damage of the patient in case 1.

A 2-year-old, spayed female, and mixed breed dog presented for assessment and treatment of severe orofacial wounds deemed to be the result of contact with corrosive materials. Physical examination revealed a large eschar covering a wound on the maxillary and nasal bones. The wound extended rostrally to the level of the canine tooth and the patient was devoid of most of the left superior labia. The left labial commissure was intact. The wound extended across the nasal bones to the right side at the level of the medial canthus with evidence of a right maxillonasal fistula (Figures 8A,B).

The patient was placed under general anesthesia for a head CT. A diagnosis of chronic osteomyelitis of the maxilla, zygomatic and nasal bones bilaterally with right maxillonasal fistula, rhinitis, and extensive loss of left dorsal subcutaneous tissue and remodeling of the right soft tissues was made (Figures 8C,D). The left maxillary premolars (205–208) were extracted due to severe periodontal attachment loss. Additionally, root canal treatment was performed on 104 and 204 due to evidence of non-vitality. The patient recovered uneventfully from general anesthesia and definitive reconstructive surgery was scheduled for a later date.

**Figure 9:**
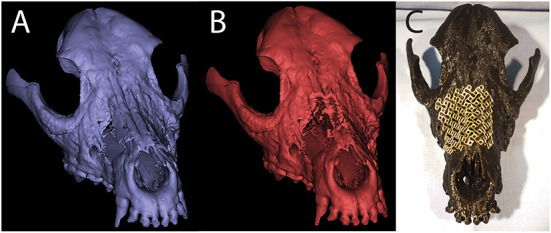
Pre- **(A)** and post- **(B)** virtual osteotomy 3D computer models for case 1. The post-virtual osteotomy model was printed and used for titanium mesh contouring, which can be seen in the photograph of the 3D printed skull **(C)**.

The CT scan of the patient was saved in DICOM format and imported into an image processing software program (Mimics, Materialize, Leuven, Belgium). Segmentation by thresholding was used to select the HU range representing mineralized tissue. A 3D model of the maxilla was created and saved as an STL file (Figure 9A). Virtual ostectomy was performed to remove segments of maxillary and nasal bone deemed to be necrotic based on CT and physical examination findings. This resulted in a new 3D model reflecting the anticipated osseous defect to be repaired in live surgery (Figure 9B). Both pre- and post-virtual surgery models were saved as an STL files and printed using an FDM 3D printer (Prusa i3, Prusa Research). A titanium mesh (matrixNeuro reconstruction mesh, DePuy Synthes) was cut and contoured to the post-virtual surgery printed model to reconstruct the anticipated osseous defect (Figure 9C).

**Figure 10:**
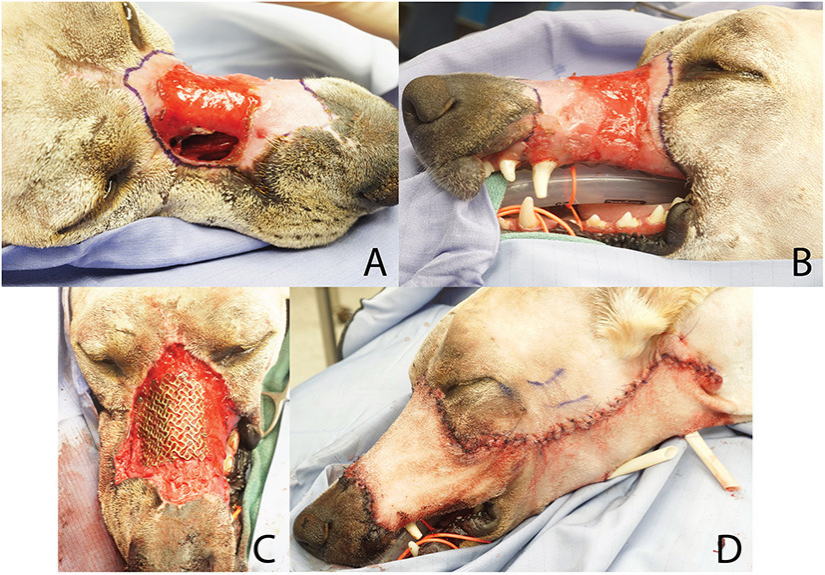
Clinical photographs of reconstruction procedure in case 1. Intra-operative debridement of wound edges **(A,B)** was followed by placement of the pre-contoured titanium mesh implant **(C)** and soft tissue closure with an angularis oris axial pattern flap **(D)**.

One month following the initial CT scan the patient was placed under general anesthesia and remaining eschar was grossly debrided followed by standard surgical preparation of the wound. Additionally, the left side of the face and neck was clipped and surgically prepared for an angularis oris axial pattern flap (39), In surgery, marked soft tissue wound margins including granulation and scar tissue were surgically excised to create a healthy recipient bed for soft tissue reconstruction (Figures 10A,B). The devitalized bone fragments were removed with a combination of surgical rongeurs and piezotome instrumentation (Piezotouch, Medtronic, Dublin, Ireland) using the printed model as a guide. A commercial freeze-dried fascia graft (Veterinary Transplant Services, Kent, USA) was secured to the nasal aspect of the maxillonasal fistula using bone tunnels and sutures to the nasal mucosa to encourage nasal mucosal re-epithelialization. The pre-contoured titanium mesh was fixed in place with four titanium cortical screws (Figure 10C). Finally, the soft tissue defect over the left and right maxillae was reconstructed with an angularis oris axial pattern flap (Figure 10D) (30, 31). The patient was evaluated at 6 and 18 months post-surgery with a CT scan performed at 6 months. The implant appeared well integrated into new fibro-osseous tissue and the patient had normal respiratory and masticatory function with no signs of rhinitis.

### Case 2–3D printed unaffected mirror image of anatomy for implant pre-contouring

**Figure 11:**
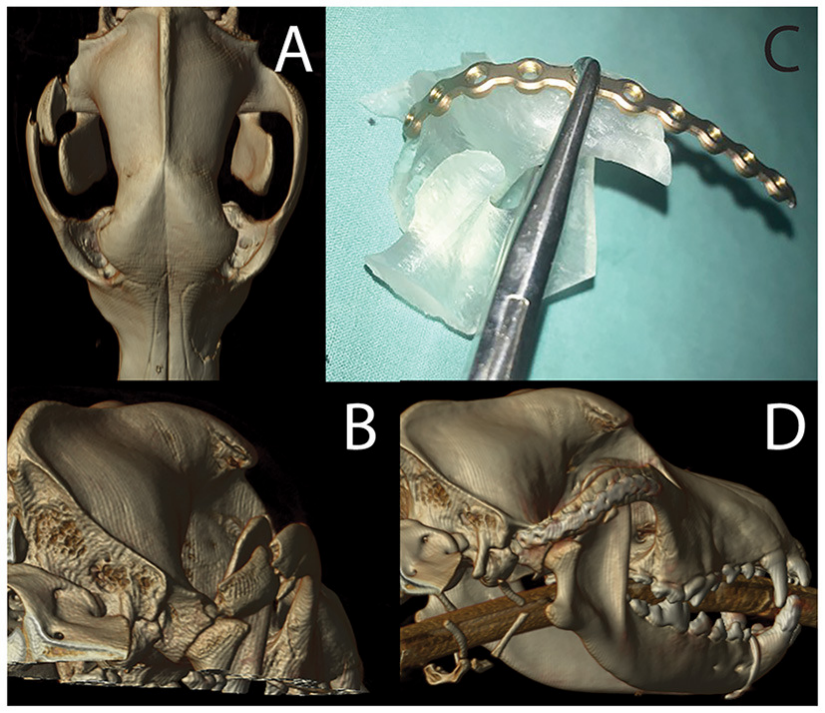
Volumetric rendering of dorsal **(A)** and caudal **(B)** views revealing the fracture segment in case 2. The photograph of mirror image 3D printed temporomandibular apparatus **(C)** was used for implant recontouring. A volumetric rendering of the post-operative CT study reveals excellent implant placement and good fracture alignment **(D)**.

An 8-month-old, intact male, Labrador retriever presented for evaluation and management of maxillofacial trauma after being hit by a car. After he was deemed to be stable by the emergency and critical care team, a head CT was performed. A segmental fracture within the right zygomatic arch was observed. This fracture extended through the rostral aspect of the zygomatic process of the temporal bone and the caudal aspect of the zygomatic bone perpendicular to the temporozygomatic suture. The fracture extended to the right temporomandibular joint with a short, oblique, comminuted fracture of the articular surface and the squamous portion of the temporal bone (Figures 11A,B). The patient was recovered from anesthesia allowing 24 hours for VSP and 3D printing. The DICOM file was uploaded into Mimics (Materialize, Leuven, Belgium) where an STL of the ROI was isolated using segmentation techniques described above. This included the temporomandibular apparatus (TMA) encompassing the entirety of the segmental fracture. Additionally, an STL mirror image of the contralateral TMA was created to be used as a template for implant contouring prior to surgery. Both the fractured TMA and the mirrored contralateral, intact TMA were printed using an SLA printer (Form3B, Formlabs, USA). By contouring the surgical implant prior to surgical placement (Figure 11C), the segmental fracture could be restored to normal anatomic orientation of the TMA, a space that is challenging to access, decreasing intra-operative time and helping to ensure a healthy articulation with decreased likelihood of TMJ ankylosis.

A skin incision was made following the ventral border of the right zygomatic arch and extended caudally to region dorsal to the temporomandibular joint. The incision was continued through the subcutaneous tissue as well as the platysma muscle. The periosteum was incised to facilitate ventral elevation of the masseter muscle at the point of insertion on the zygomatic arch. This exposed the fractured segment of the zygomatic arch and the temporal bone. The segmental fracture of the zygomatic arch was manually reduced followed by application of the pre-contoured titanium miniplate. The implant was secured to the rostral and caudal segments and the comminuted segment was secured to the implant. The most caudal screw hole on the temporal bone was not accessible and was left empty. The surgical wound was closed in three layers followed by post-operative CT imaging to verify acceptable reduction (Figure 11A).

Two week following surgery, the patient returned for skin suture removal and conscious oral evaluation. The owners reported that he was eating well and demonstrated what was considered to be normal ability to use his mouth. Six week following surgery, a follow-up CT scan was performed. This imaging revealed progressive healing of the fracture.

### Case 3—VSP

**Figure 12:**
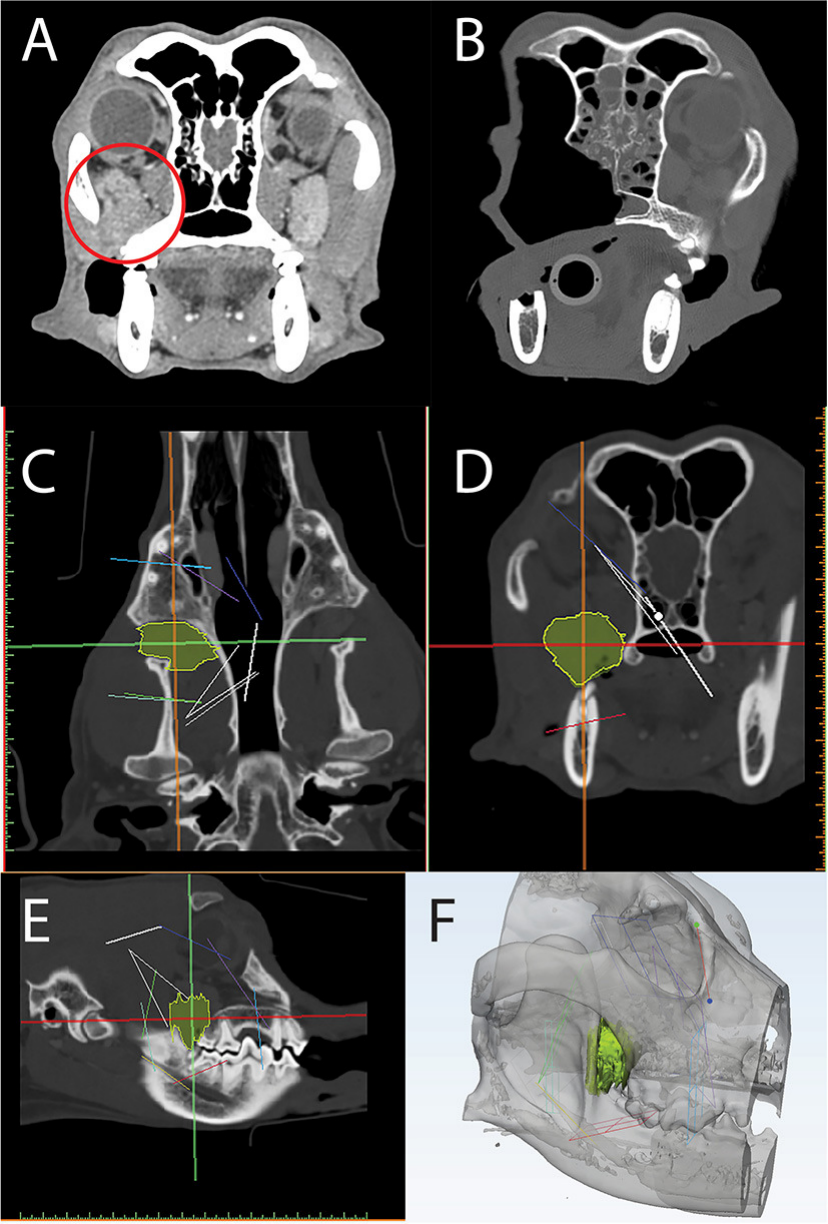
Axial CT of a post-contrast image study depicting a caudal maxillary neoplasm in case 3 **(A)**. Panels C-F are computer screenshots depicting the segmentation of the neoplasm in yellow **(C–E)** and VSP on a 3D computer model **(F)**. The excised segment can be appreciated in the immediate post-operative axial CT image **(B)**. Note the medial orbitectomy plane is consistent with virtual surgical plan.

An 8-year-old, spayed female, vizsla presented for treatment of an osteosarcoma located inferior to the right eye. On presentation, the oral mass measured 2.7 cm in length and was firm and non-mobile. Fine needle aspirates of the mandibular lymph nodes revealed no evidence of local metastasis. Head CT revealed a 3.2 x 3.2 x 3.5 cm osteolytic and osteoproliferative soft tissue and mineral attenuating, moderately contrast enhancing mass within the right caudal maxilla (Figure 12A). The mass extended caudally into the right pterygopalatine recess and orbital space causing dorsolateral displacement of the right globe and caudoventral displacement of the right zygomatic salivary gland. The mass extended rostrally and ventrally to include the alveolar bone associated with the previously extracted right maxillary fourth premolar tooth.

The DICOM data was used to create a computer model of the skull through an automated thresholding process (Materialize, Leuven, Belgium). Using a manual approach, the tumor was also segmented (Figures 12C–E) and the STL file of the skull and tumor was imported into a design software (3-Matic, Materialize, Leuven, Belgium) to facilitate VSP. Intended surgical margins were mapped in several planes to define the ostectomy sites (Figure 12F).

The CT scan and STL files were opened on a monitor within the operatory for reference throughout the surgical procedure. The tumor was resected en bloc, including the orbitozygomaticomaxillary complex (OZMC) and associated musculature, oral mucosa, gingiva, and dentition utilizing a combined intra- and extraoral approach with a transpalpebral exenteration. The resection including portions of the caudal maxilla, lacrimal, frontal, palatine, zygomatic, temporal, and pterygoid bones were performed as outlined in the VSP and confirmed on the post-operative CT scan (Figure 12B). At 2 week and 6 month follow up examination, all surgical wounds were healed.

### Case 4—VSP, 3D printed patient model, 3D printed patient-specific implant

**Figure 13:**
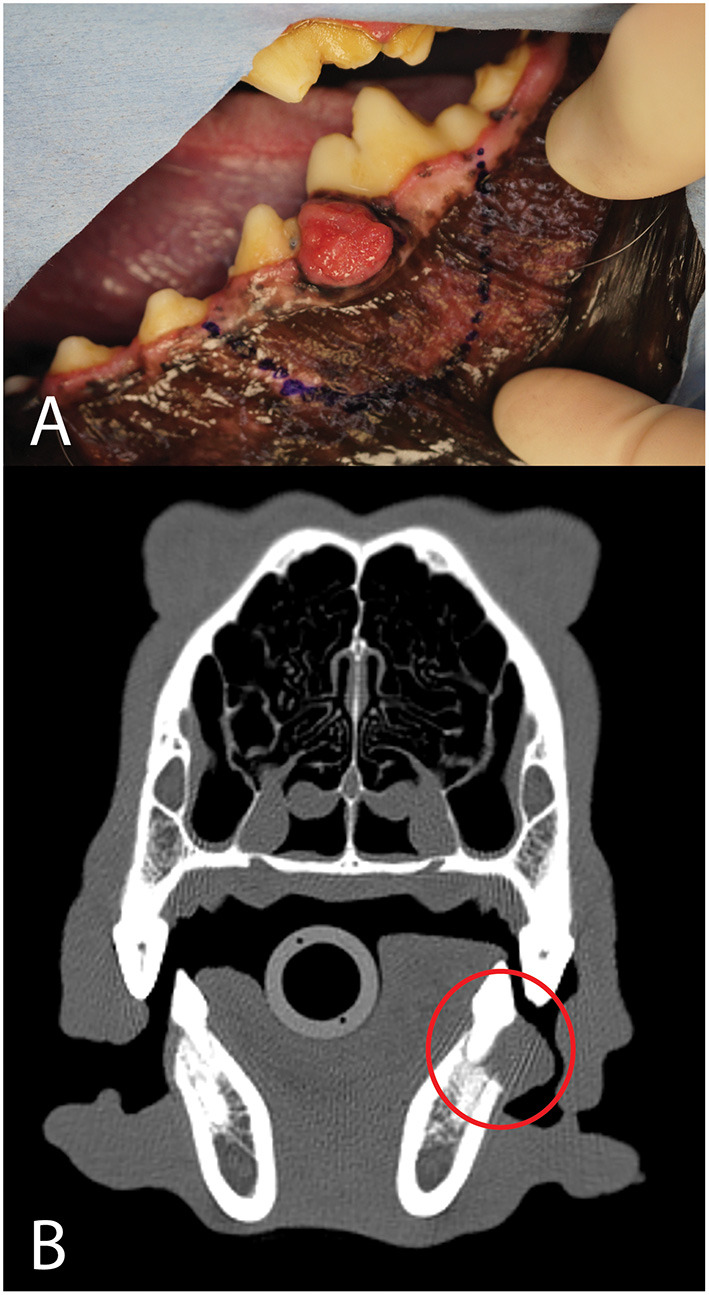
Photograph **(A)** and axial CT image **(B)** of the neoplasm (red circle) in case 4.

A 7-year-old, male castrated, golden retriever was referred for treatment of a previously diagnosed canine acanthomatous ameloblastoma (Figure 13). The owners elected a segmental mandibulectomy with immediate mandibular reconstruction. Reconstruction options discussed with the owner included (1) reconstruction with a standard titanium mandibular reconstruction plate with bone graft and (2) reconstruction using a patient-specific 3D printed titanium plate with an integrated titanium lattice basket designed for supporting bone graft material and osseointegration. The owners opted for the latter, as this has been demonstrated to come with a high success rate in human mandibular reconstruction surgery (31, 35).

**Figure 14:**
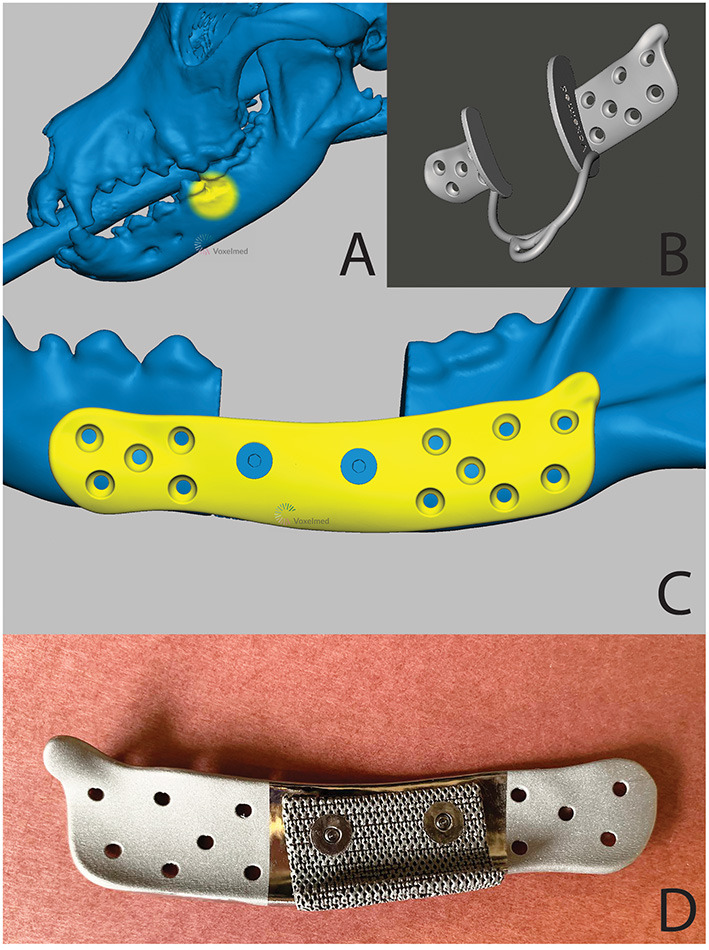
A 3D computer model of the skull **(A)**—tumor location is highlighted in yellow), the patient-specific surgical saw guide **(B)** and patient-specific implant **(C)**—the countersunk screws to attach mesh structure are highlighted in blue) of the patient in case 1. **(D)** is a pre-operative photograph of the patient-specific implant from the medial aspect. Reprinted from Thatcher GP, Soukup JW. Virtual surgical planning and 3D printing in veterinary dentistry and oromaxillofacial surgery. *Vet Clin North Am Small Anim Pract*. 2022; 52(1):221-234 with permission from Elsevier.

Head CT was performed and the DICOM data was sent to a third-party bioengineering company (Voxelmed, Germersheim, Germany) that specializes in design solutions for veterinary orthopedic and oncologic reconstruction. The DICOM data was imported into and open-source image processing software (Invesalius, Campinas, Brazil) and segmented by a thresholding technique. The region grow function was used to extract the left mandible from the entire data set. This mandible was saved as an STL file. A measurement tool was used to map out 1 cm circumferential margins around the radiographically identified tumor (Figure 14A). This STL was imported into a design software (FreeForm Plus, 3D-Systems, Rock Hill, USA) where three parts were designed. First, a mirror reconstruction technique was employed to merge the healthy bone of the right mandible onto the left mandible with the tumor resected. This healthy mandible was used for implant design by marking and extracting the outer region resulting in a patient specific 1.6 mm thick mandibular reconstruction plate, designed to fit the detailed anatomic contours of the mandible (Figure 14C). A Boolean subtraction function was used to strategically place countersunk screw holes in regions that would minimize risk of damage to the neurovasculature and tooth roots. At this point, in consultation with the OMF surgeon, the engineer made modifications to the design so that it would have a lower ventrodorsal profile and screw size was verified. Once the plate design was finalized, an osteotomy guide was designed with saw blade guides oriented perpendicular to the mandibular body and including drill cylinders for Kirshner wires (Figure 14B). Finally, the segment to be removed was used as a template for the titanium lattice scaffold. This porous scaffold was designed to be fixed by set screws to the inner surface of the patient specific mandibular reconstruction plate (Figure 14D). After approval of the design by the veterinary OMFS team, the final STL files were ready for fabrication. The titanium alloy parts, including the mandibular reconstruction plate, the osteotomy guide and the lattice were fabricated using a selective laser melting (SLM) printer (Brand name, manufacturer). After the SLM build, the parts were cooled, and the surfaces of reconstruction plate underwent post-processing. The inner surface, to be in contact with bone was sandblasted and the outer surface was machined using laser ablation to achieve a smooth finish. The STL file of the mandible was printed with an FDM printer (Prusa i3, Prusa Research) and another copy of the osteotomy guide was printed with an SLA printer (Form2, Formlabs) for surgical rehearsal. The titanium mandibular implants and osteotomy guide along with the models were sterilized prior to surgical treatment.

**Figure 15:**
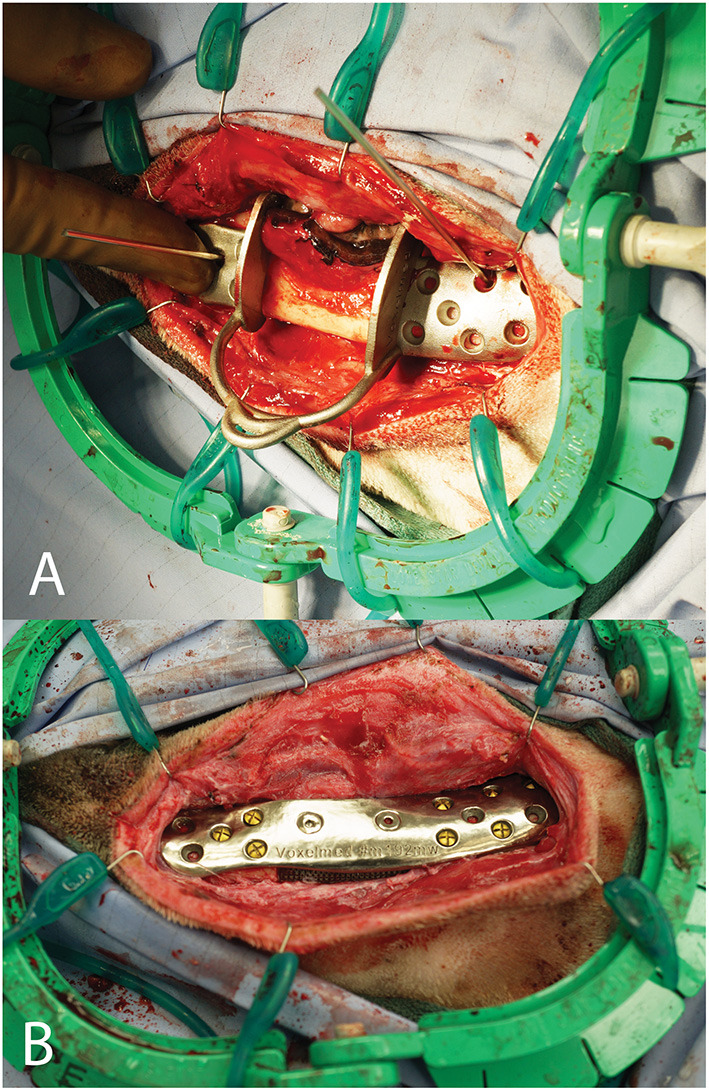
Intra-operative photograph of the patient-specific surgical saw guide held in place with Kirshner wires **(A)** and of the patient-specific implant in place **(B)**.

The patient was routinely placed under general anesthesia and the teeth were scaled and polished in the dental operatory. The intra-oral intended surgical margins were marked with a surgical pen. An appropriate surgical field exposing the mandible was clipped and prepared. In addition, the left proximal humerus was prepared for autologous, cancellous bone graft harvesting. A final skin preparation was performed, and the patient was appropriately draped. The greater tubercle of the proximal humerus was palpated, and an autogenous cancellous bone graft was obtained using standard techniques. The pre-marked oral soft tissue margins were incised using a standard intra-oral approach followed by an extra-oral, ventral approach to the left mandible. Following reflection of the muscles and periosteum from the mandible, the osteotomy guide was secured in place with Kirshner wires placed in the rostral and caudal segments of the osteotomy guide (Figure 15A). An oscillating saw was used to perform the segmental mandibulectomy followed by ligation of the vessels in the mandibular canal. The oral soft tissues were closed through the extra-oral surgical field, followed by flushing with copious amounts of sterile saline. The osteotomy guide was lifted off the pre-placed Kirshner wires and the patient-specific mandibular reconstruction plate with the lattice graft basket secured, was placed using the corresponding holes. The plate was secured to the rostral and caudal mandible segments with pre-measured titanium cortical screws (Synthes, Paoli, PA) (Figure 15B). Finally, the bone graft was packed into the titanium lattice on the lingual aspect and the surgical site was closed in 3 layers. The patient recovered from anesthesia uneventfully and returned home the following day. No complications were noted at the time of follow-up at 2 weeks. At 1-year post-op, mucosal erosion over the implant was noted and a debridement and closure procedure were performed. Two months later, due to concern of infection, surgical debridement was performed, and the patient-specific implant was replaced with a 2.4 titanium reconstruction plate. Six months later, all hardware was removed.

## Conclusion

Given the increasing expectations for patient-specific oromaxillofacial treatments from clients and surgeons alike, advances in OMFS are inevitable. Integration of VSP and 3D printing into the OMF surgeon's armamentarium will likely lead to decreased surgical times, improved patient outcomes and advanced surgeon skill set. Despite some disadvantages and limitations discussed, we feel the overall balance supports increased incorporation of advanced 3D techniques and printing into OMFS. Veterinary OMFS teams are among those specialties leading the integration of these techniques into clinical practice.

## Data availability statement

The original contributions presented in the study are included in the article/supplementary material, further inquiries can be directed to the corresponding author.

## Ethics statement

Written informed consent was obtained from the owners for the publication of any potentially identifiable images or data included in this article.

## Author contributions

JK and GT: conception and design of project, manuscript drafting and revision. JB: manuscript drafting and revision. JS: conception, design and supervision of project, manuscript drafting and revision. All authors contributed to the article and approved the submitted version.

## Conflict of interest

The authors declare that the research was conducted in the absence of any commercial or financial relationships that could be construed as a potential conflict of interest.

## Publisher's note

All claims expressed in this article are solely those of the authors and do not necessarily represent those of their affiliated organizations, or those of the publisher, the editors and the reviewers. Any product that may be evaluated in this article, or claim that may be made by its manufacturer, is not guaranteed or endorsed by the publisher.
